# Supplementary material for: Sequencing artifacts derived from a library preparation method using enzymatic fragmentation
Source: PLoS One. 2020 Jan 3;15(1):e0227427. doi: 10.1371/journal.pone.0227427 (PMC6941819; doi:10.1371/journal.pone.0227427)
Supplement: S1 Fig — Each panel indicates the location of SCPs filtered by the noise-canceling algorithm (x-axis) and the number of reads (y-axis) for each odd-numbered-length palindrome. Palindrome length is indicated by the number at the right shoulder of each panel. Palindromic sequences and SNVs are shown as black lines and red points, respectively. Thin and thick dashed lines indicate the end and 30 bases from edge of the read, respectively. Each read is 100 bases in length, as shown above the panel. (DOCX) [file pone.0227427.s001.docx]

S1 Fig


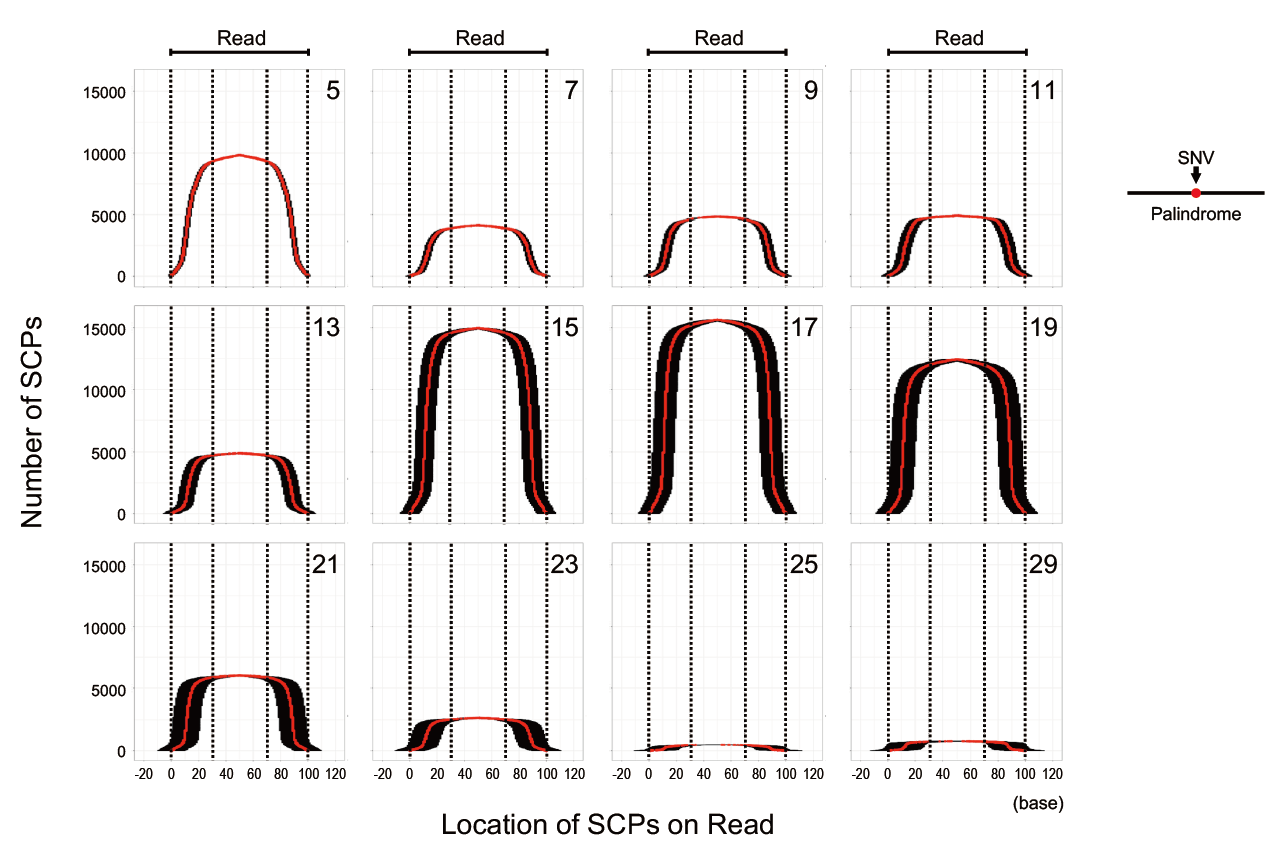


Location of SNV-centered palindromes (SCPs) on the read. Each panel indicates the location of SCPs filtered by the noise-canceling algorithm (*x*-axis) and the number of reads (*y*-axis) for each odd-numbered-length palindrome. Palindrome length is indicated by the number at the right shoulder of each panel. Palindromic sequences and SNVs are shown as black lines and red points, respectively. Thin and thick dashed lines indicate the end and 30 bases from edge of the read, respectively. Each read is 100 bases in length, as shown above the panel.
